# Impact of a nurses' protocol-directed weaning procedure on outcomes in patients undergoing mechanical ventilation for longer than 48 hours: a prospective cohort study with a matched historical control group

**DOI:** 10.1186/cc3030

**Published:** 2005-01-17

**Authors:** Jean-Marie Tonnelier, Gwenaël Prat, Grégoire Le Gal, Christophe Gut-Gobert, Anne Renault, Jean-Michel Boles, Erwan L'Her

**Affiliations:** 1 Réanimation Médicale, Centre Hospitalier Universitaire de la Cavale Blanche, Brest, France

**Keywords:** intensive care unit, mechanical ventilation, protocol-directed weaning

## Abstract

**Introduction:**

The aim of the study was to determine whether the use of a nurses' protocol-directed weaning procedure, based on the French intensive care society (SRLF) consensus recommendations, was associated with reductions in the duration of mechanical ventilation and intensive care unit (ICU) length of stay in patients requiring more than 48 hours of mechanical ventilation.

**Methods:**

This prospective study was conducted in a university hospital ICU from January 2002 through to February 2003. A total of 104 patients who had been ventilated for more than 48 hours and were weaned from mechanical ventilation using a nurses' protocol-directed procedure (cases) were compared with a 1:1 matched historical control group who underwent conventional physician-directed weaning (between 1999 and 2001). Duration of ventilation and length of ICU stay, rate of unsuccessful extubation and rate of ventilator-associated pneumonia were compared between cases and controls.

**Results:**

The duration of mechanical ventilation (16.6 ± 13 days versus 22.5 ± 21 days; *P *= 0.02) and ICU length of stay (21.6 ± 14.3 days versus 27.6 ± 21.7 days; *P *= 0.02) were lower among patients who underwent the nurses' protocol-directed weaning than among control individuals. Ventilator-associated pneumonia, ventilator discontinuation failure rates and ICU mortality were similar between the two groups.

**Discussion:**

Application of the nurses' protocol-directed weaning procedure described here is safe and promotes significant outcome benefits in patients who require more than 48 hours of mechanical ventilation.

## Introduction

The duration of weaning from mechanical ventilation (MV) represents a large proportion of the overall ventilation period [1]. The time from initiation of weaning to successful endotracheal extubation may account for as much as 40% of the overall ventilatory time [1]. A great number of studies have demonstrated that prompt recognition of reversal of respiratory failure using standardized procedures and daily screening may shorten the overall duration of MV [2-5]. However, those studies were mainly conducted by respiratory therapists in North American intensive care units (ICUs), whereas in Europe the respiratory therapist's roles are mainly assumed by nurses and physicians. Despite the accumulating evidence to support their routine use, the value of standardized weaning procedures continues to be of clinical and academic interest [6].

In October 2001, the French intensive care society (Société de Réanimation de Langue Francaise) organized a consensus conference to develop recommendations for MV weaning in the ICU [7]. The aim of the present prospective cohort study, which included a matched historical control group, was to determine whether a routine nurses' protocol-directed weaning procedure based on these recommendations and daily screening were efficient in terms of MV duration and ICU length of stay (LOS) in patients who required more than 48 hours of MV.

## Methods

### Study design

This prospective study was conducted in the 12-bed ICU of an 800-bed teaching hospital from January 2002 to February 2003. We employed a matched control group (1:1 matching) of patients identified from a historical database.

### Selection of patients and controls

Screened patients were those who required more than 48 hours of MV and who satisfied eligibility criteria (see below) for a spontaneous breathing trial (SBT). Exclusion criteria were tracheostomy before ICU admission or within the first 48 hours of ICU management, and age under 18 years.

A computer-generated list of potential control individuals was obtained from a historical database of patients attending the same ICU (977 patients from 1999 to 2001). Controls were selected based on matching in terms of the following criteria: age (±5 years), sex, Simplified Acute Physiology Score (SAPS) II (±5 points; calculated within the first 24 hours of ICU admission), and admission diagnosis. The list of potential controls was reviewed for the best possible match, giving a ranking priority to SAPS II, followed by age, sex and then diagnosis.

### Study protocol

**Figure 1:**
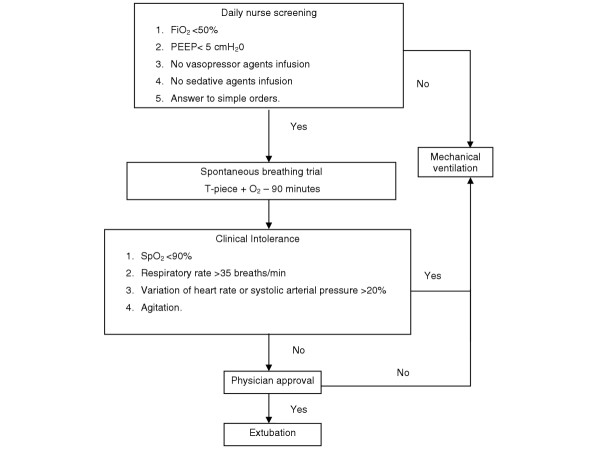
Mechanical ventilation weaning protocol. Daily nurse screening identified patients eligible for weaning. A spontaneous breathing trial was considered to be successful when the patient could breathe spontaneously for 90 min without clinical intolerance. For such patients, physicians were then asked to approve discontinuation of mechanical ventilation. If the spontaneous breathing trial was not tolerated, then the patient was returned to their prior ventilator settings and screened the day after. FiO_2_, fractional inspired oxygen; PEEP, positive end-expiratory pressure; SpO_2_, pulse oximetry.

The study protocol was written according to the final recommendations of the Société de Réanimation de Langue Francaise's consensus conference on weaning. Patient eligibility for the weaning procedure was identified by daily screening by nurses. Screening was deemed to start immediately after ICU admission. The eligibility criteria for a SBT were the following: fractional inspired oxygen <50%; positive end-expiratory pressure <5 cmH_2_O; no vasopressor infusion; no sedative agent infusion; and response to simple orders. Physician approval for initiation of SBT was not required. A planned SBT duration of 90 min was employed, and SBTs were always performed using a T-piece. The SBT was terminated before 90 min had elapsed and considered a failure if any of the following criteria were satisfied: pulse oximetry <90%, a respiratory rate >35 breaths/min, a heart rate or a systolic arterial pressure variation >20%, or occurrence of patient agitation. All of these criteria for failure were specifically recorded. A SBT was considered to be successful when the patient could breathe spontaneously for 90 min. Physicians were asked to approve discontinuation of MV following a successful SBT. Extubation was therefore performed if cough was subjectively considered efficient, and if a leak test was considered positive (inspiratory and/or expiratory air leaks after cuff deflation). If the SBT was not well tolerated, then the failure criteria were specifically recorded and the patient returned to their prior ventilator settings and mode. Such patients were then subjected to screening the following day (Fig. 1).

### Pre-protocol weaning management (historical controls)

Before establishment of the nurse's protocol-directed weaning procedure, routine weaning from MV was 'physician directed', as in most European ICUs. Physician's individual preferences determined the mode of weaning (volume assisted controlled, pressure support ventilation, or T-piece). Weaning criteria were not monitored daily. The decision to extubate after a successful weaning procedure was also physician directed.

### Definitions

All definitions were selected *a priori *before study analysis. Admission diagnosis was divided into four main groups: medicine, surgery, neurosurgery and acute exacerbations in chronic obstructive pulmonary disease. Ventilator-associated pneumonia was defined as the initiation of antibiotics and at least two of the following criteria: positive protected bronchoscopy cultures; fever or rising leucocyte count; and characteristic chest radiograph findings. The duration of ventilation was calculated as follows: (day of extubation + 1) - (day of intubation). Successful discontinuation of MV was defined as continuous independence from ventilator support for a period of at least 48 hours. Unsuccessful MV discontinuation was defined as need for noninvasive ventilation and/or reintubation within a 48 hour period. Criteria to initiate noninvasive ventilation following extubation were occurrence of clinical signs of acute respiratory failure, with or without hypercapnia.

### Outcomes

The overall duration of MV and the ICU LOS were considered primary outcomes. Secondary outcomes were the overall incidence of ventilator-associated pneumonia, unsuccessful extubation rates and ICU mortality.

### Statistical analysis

Categorical variables were expressed as percentage and continuous variables as mean ± standard deviation. *P *< 0.05 was considered statistically significant. Percentages were compared using χ^2 ^tests, and means using Student's t-test. Kaplan–Meier curves were used to determine the probability of remaining ventilated during the overall ICU LOS; curves were compared using the log-rank test. When patients died before discontinuation of MV, matched data were censored within the analysis.

## Results

### Physiological variables

**Figure 2:**
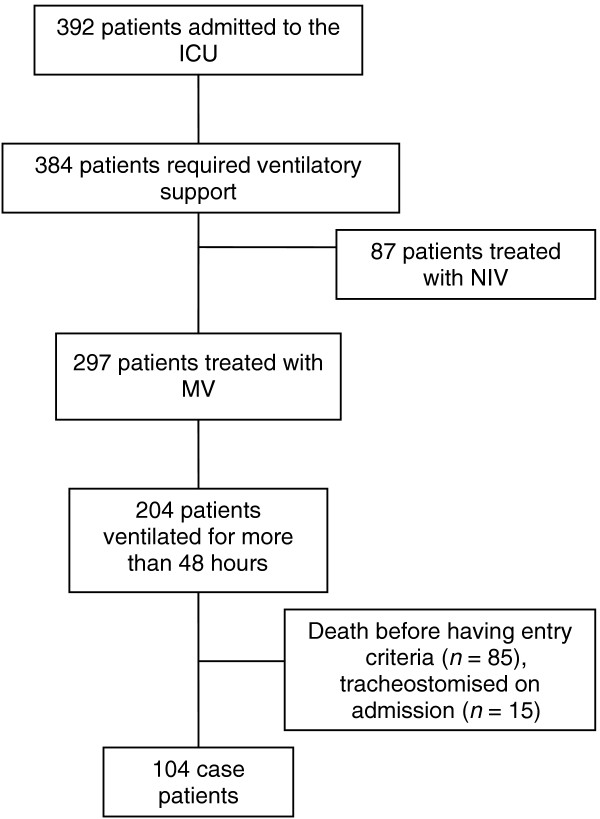
Case selection. ICU, intensive care unit; NIV, noninvasive ventilation.

During the prospective study period 392 patients were admitted to our ICU, of whom 384 patients required ventilatory support. A total of 297 patients were mechanically ventilated through an endotracheal tube, of whom 204 required MV for longer than 48 hours. Among these 204 eligible patients, 100 were excluded from analysis because they died prior to initiation of any weaning procedure (85 patients) or because they were tracheostomized before admission or within the first 48-hour period (15 patients; Fig. 2). After 1:1 matching, 208 patients were finally included in the analysis: 104 patients in the prospective routine nurse's protocol-directed weaning procedure (cases) and 104 patients in the standard physician-directed weaning procedure (controls). Matching was successful for all parameters, and patient demographic variables were similar within groups (age 56 ± 18 years, sex ratio [male/female] 3/2; SAPS II = 49 ± 18). Admission diagnoses were similarly distributed within groups (Table 1).

### Duration of mechanical ventilation and intensive care unit length of stay

**Figure 3:**
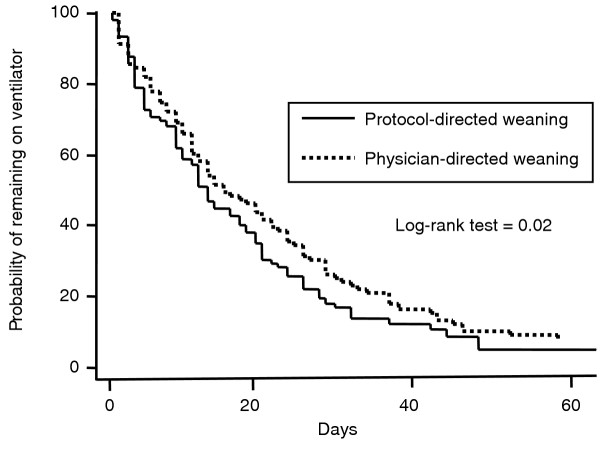
Kaplan–Meier curves of the risk for remaining mechanically ventilated in protocol-directed (cases) versus physician-directed weaning groups (controls). The protocol-directed weaning procedure allowed reduction in the overall duration of mechanical ventilation, whatever the patient's diagnosis. The overall mechanical ventilation duration was 16.6 ± 13 days in cases and 22.5 ± 21 days in controls (*P *= 0.02).

All patients were under volume-assisted controlled or pressure support ventilation before entering the weaning procedure. The overall MV duration was 16.6 ± 13 days within cases, and 22.5 ± 21 days within controls (*P *= 0.02); this difference between groups is illustrated by Kaplan–Meier analysis in Fig. 3. The ICU LOS was 21.6 ± 14.3 days within cases and 27.6 ± 21.7 days within controls (*P *= 0.02). In subgroup analysis, the duration of MV and ICU LOS were shorter for medical patients (Table 2).

### Outcomes

No significant differences in unsuccessful MV discontinuation rates were observed between groups (31% for cases versus 35% for controls; *P *= 0.81). Mortality was similar between the two groups (7% for cases versus 5% for controls; *P *= 0.92). A positive trend toward a decrease in ventilator-associated pneumonia rate was observed for the cases (20.2% versus 31%; *P *= 0.12; Table 3).

## Discussion

The present study was designed to determine the clinical benefit of a nurse's protocol-directed weaning procedure in a broad range of ICU patients (including both medical and surgical patients) who had not been disconnected from the ventilator after 48 hours of MV. Our findings demonstrate the effectiveness of such a procedure performed on a routine basis. The nurse's protocol-directed weaning procedure reduced the duration of MV and the overall ICU LOS in patients who remained dependent on MV after a 48-hour period, without any increase in adverse events or in rate of unsuccessful extubation. Easily implemented, this protocol-directed weaning procedure synthesizes an approach that incorporates the following: daily screening by nursing staff and a single daily 90 min SBT with T-piece trial in selected patients. The criteria used in this procedure to screen patients for readiness for the weaning trial were very simple clinical criteria.

Some authors have demonstrated a key role for daily screening using predetermined criteria during the weaning process [2,4]. In both of those studies, patients who satisfied the criteria were subjected to a 2 hour trial of CPAP while they remained attached to the mechanical ventilator circuit. How exactly a SBT should be performed remains subject to debate, and its optimal duration is not known, although there are data suggesting that it may be shortened [8-11]. For the routine procedure, we arbitrarily chose a 90 min duration and to perform the trial while the patient was disconnected from the ventilator, without adding any positive end-expiratory pressure.

The mean MV duration and ICU LOS found in this study differ significantly from those of previous studies [2,3,12]. In the study by Ely and coworkers [2] patients received MV for a median of 4.5 days, and in the study by Esteban and coworkers [10] patients were ventilated for a median of 5 days. Moreover, in the study by Ely and colleagues the ICU LOS was similar between groups. The main reasons for such a difference between our study and the previous ones are the higher mean SAPS II in our patients (49 ± 18 in both groups) and the selection of patients who survived and were not weaned from MV after a 48 hour period (i.e. patients whose conditions probably remained unstable for a long period and/or those who may be considered 'difficult to wean'). Therefore, we believe that we selected patients who may derive particular benefit from a decrease in the duration of MV.

The unsuccessful extubation rate also appears rather high in our patients as compared with previous studies (31% within cases versus 35% within controls) [10,13-15]. However, this finding must be interpreted with caution because we considered unsuccessful extubation to be the need for any form of ventilatory assistance within a 48 hour period (either delivered noninvasively via face mask or invasively via an endotracheal tube), whereas in the vast majority of other studies unsuccessful extubation was considered just as need for endotracheal intubation. If we consider the reintubation rate alone, the incidence of extubation failure decreases to 21% in cases and 18% in controls. However, one may observe that, apart from earlier extubation, the failure rates were similar between the two groups (i.e. patients were not withdrawn too early, as compared with the 'standard' procedure).

Apart from the rather high mean SAPS II levels, the overall mortality rate in our study appears rather low in both groups (5%). However, in accordance with the analysis protocol we excluded patients who died before the predetermined weaning criteria were satisfied, and so this finding cannot be considered to be an adequate reflection of overall ICU mortality rate.

Case series with historical controls have advantages over randomized trials when the effects of routine daily procedures are studied. Indeed, it is easier for a broad range of nursing staff to institute a protocol throughout an entire ICU population than it is to limit it to specific monitored patients. Moreover, there is no risk for crossover effect, by which the staff may modify their behaviour toward control patients and thus blur the distinction between groups.

On the other hand the nonrandomized design of our study is a major limitation. First, only randomization can ensure comparability between cases and controls. Even if cases and controls were matched for age, sex, SAPS II and diagnosis on admission, selection bias cannot be fully excluded and might account for part of our results. Second, evolution of medical care during the study period might have influenced our results [16]. However, the use of sedatives and paralytics, and routine patient positioning and ventilatory settings did not fundamentally change over the period of interest (1999–2003). For example, patients with acute respiratory distress syndrome have been ventilated using a small tidal volume and an 'open lung approach' since publication of the study by Amato and coworkers [17].

## Conclusion

This study demonstrates that a routine and simply applied nurse's protocol-directed weaning procedure was safe and promoted major clinical and outcome benefits for patients requiring MV over a 48 hour period. These benefits were obtained without increasing the average number of complications or the rate of unsuccessful extubation.

## Key messages

• 	A simply applied nurse's protocol-directed weaning procedure was safe and promoted major clinical and outcome benefits for patients.

• 	Protocol-directed weaning procedure should be used in all ICUs.

## Abbreviations

ICU = intensive care unit; LOS = length of stay; MV = mechanical ventilation; SAPS = Simplified Acute Physiology Score; SBT = spontaneous breathing trial.

## Competing interests

The author(s) declare that they have no competing interests.

## Authors' contributions

JMT drafted the manuscript and participated in the design of the study. GP conceived the study and helped to draft the manuscript. GLG performed the statistical analysis. CGG collected data. AR colected data. JMB participated in the design of the study. EL participated in its design and coordination, and helped to draft the manuscript. All authors read and approved the final manuscript.

## Figures and Tables

**Table 1:** Baseline characteristics in the study patients

Physiological parameters	Protocol-directed weaning group (cases)	Physician-directed weaning group (controls)
Patients (*n*)	104	104
Male sex (*n *[%])	62 (60)	62 (60)
Age (years; mean ± SD)	57 ± 18	56 ± 18
SAPS II (mean ± SD)	49 ± 18	49 ± 18
Admission diagnosis (*n *[%])		
Medicine	54 (52)	63 (61)
Surgery	25 (24)	20 (19)
Neurosurgery	19 (18)	16 (15)
COPD	6 (6)	5 (5)

Matching was successful for all parameters, and patients' physiological parameters were similar between groups. Admission diagnoses were similarly distributed between groups. COPD, chronic pulmonary obstructive disease; SAPS, Simplified Acute Physiology Score; SD, standard deviation.

**Table 2:** Outcome comparison between the study groups, according to admission diagnosis

Outcomes	Protocol-directed weaning group (cases)	Physician-directed weaning group (controls)	*P*
Mechanical ventilation duration (days)			
Medicine	15.3 ± 13	23 ± 22.6	0.02
Surgery	19.1 ± 15.2	17.4 ± 13.7	0.72
Neurosurgery	18.9 ± 12.8	26.3 ± 23.1	0.29
COPD	14.1 ± 14.6	24.9 ± 24	0.33
Combined	16.6 ± 13	22.5 ± 21	0.02
ICU length of stay (days)			
Medicine	20.1 ± 13.7	27.7 ± 23	0.02
Surgery	25 ± 17	23.3 ± 15.4	0.72
Neurosurgery	23 ± 13	34 ± 24	0.09
COPD	20.4 ± 14.6	28.5 ± 25	0.47
Combined	21.6 ± 14.3	27.6 ± 21.7	0.02

Values are expressed as mean ± standard deviation. COPD, chronic pulmonary obstructive disease; ICU, intensive care unit.

**Table 3:** Comparison of complications between study groups

Complications	Protocol-directed weaning group	Physician-directed weaning group	*P*
Unsuccessful MV discontinuation	33 (31)	37 (35)	0.81
Reintubation within 48 hours	22 (21)	19 (18)	0.33
NIV for postextubation respiratory distress	22 (21)	26 (25)	0.15
Ventilator-associated pneumonia	21 (20)	33 (31)	0.12
ICU mortality	7 (7)	5 (5)	0.92

Values are expressed as number (%). No significant differences between groups were observed. ICU, intensive care unit; MV, mechanical ventilation; NIV, noninvasive ventilation.
